# Anti-Amnesic Effects of Epigallocatechin Gallate on Scopolamine-Induced Learning and Memory Dysfunction in Sprague-Dawley Rats

**DOI:** 10.3390/antiox11010001

**Published:** 2021-12-21

**Authors:** Min-Jeong Kim, Eun-Sang Hwang, Kwan Joong Kim, Sungho Maeng, Ho Jin Heo, Ji-Ho Park, Dae-Ok Kim

**Affiliations:** 1 Department of Food Science and Biotechnology, Kyung Hee University, Yongin 17104, Korea; mkimella111@khu.ac.kr (M.-J.K.); Joong@khu.ac.kr (K.J.K.); 2 Department of Gerontology, Graduate School of East-West Medical Science, Kyung Hee University, Yongin 17104, Korea; eunsanghwang@khu.ac.kr; 3 Department of Comprehensive Health Science, Graduate School of East-West Medical Science, Kyung Hee University, Yongin 17104, Korea; jethrot@khu.ac.kr; 4 Division of Applied Life Science (BK21), Institute of Agriculture and Life Science, Gyeongsang National University, Jinju 52828, Korea; hjher@gnu.ac.kr; 5 Department of East-West Medicine, Graduate School of East-West Medical Science, Kyung Hee University, Yongin 17104, Korea; 6 Department of Food Innovation and Health, Graduate School of East-West Medical Science, Kyung Hee University, Yongin 17104, Korea

**Keywords:** behavioral test, epigallocatechin gallate (EGCG), green tea, hippocampus, long-term potentiation, organotypic culture

## Abstract

Epigallocatechin gallate (EGCG) is a major flavan-3-ol of green tea polyphenols that exhibits various beneficial health effects, including antioxidant, anti-bacterial, and anti-inflammatory properties. This study aimed to evaluate whether EGCG prevents scopolamine-induced learning and memory impairment in *in vivo* and *ex vivo* models. Male Sprague-Dawley (SD) rats were pre-treated with EGCG (5 mg/kg/day; intraperitoneal injection (i.p.)) for 10 days. Then, EGCG and scopolamine (1 mg/kg/day; i.p.) were applied 60 and 30 min before the behavioral tests, respectively, for another 9 days. EGCG alleviated the cognitive deficits in the Y-maze, passive avoidance, and Morris water maze tests. EGCG showed improved cholinergic functions by decreasing acetylcholinesterase activity in hippocampi dissected from the brain of the rats after the behavioral tests. EGCG also reduced oxidative stress, partly due to increased superoxide dismutase activity and decreased malondialdehyde level in the hippocampi of the rat brains after the behavioral tests. Furthermore, EGCG attenuated the scopolamine-induced blockade of long-term potentiation in organotypic hippocampal tissue of seven-day-old SD rats. Taken together, these results suggested that EGCG is a potential therapeutic agent for alleviating cognitive dysfunction.

## 1. Introduction

Alzheimer’s disease (AD) is the most prevalent age-dependent neurodegenerative disorder characterized by impairment of learning, memory, and cognitive deficits [1]. Learning and memory impairment are associated with cholinergic dysfunction due to elevated acetylcholinesterase (AChE) activity and the inhibition of acetylcholine (ACh) release in the central nervous system [2]. Thus, AChE inhibitors (e.g., donepezil, memantine, and rivastigmine) are used for symptom relief in clinical therapies for AD [3,4]. However, the therapeutic effects of these synthetic drugs are poor and have side effects such as diarrhea, vomiting, nausea, and insomnia [3]. Thus, interest in alternative drugs from natural substances with fewer side effects has increased recently.

A scopolamine model that is based on cholinergic dysfunction in the brain is widely used as an experimental animal model for AD [5,6,7]. Scopolamine, a muscarinic cholinergic receptor antagonist, interferes with the processes of learning acquisition and memory formation. In addition, many studies have reported that scopolamine also induces oxidative stress in the brain [8,9]. Oxidative stress causes irreversible apoptosis of neurons and damage to lipids, proteins, and DNA, which can lead to neurological dysfunction and then cognitive impairment. These detrimental consequences of the oxidative stress induced by scopolamine are similarly shown in the pathogenesis of AD [5]. Thus, a potential therapeutic agent that mitigates the harmful effects of oxidative stress might be useful as a potential tool for AD treatment.

Green tea is one of the most popular beverages worldwide. Green tea contains high amounts of catechins (also known as flavan-3-ols) and has many health benefits, including anti-inflammatory, antioxidant, and neuroprotective activities [10,11]. Epigallocatechin gallate (EGCG) is the most abundant flavan-3-ol among catechins of green tea [12] and is responsible for a variety of beneficial pharmacologic and physiological effects of green tea [13]. EGCG possesses two galloyl moieties and eight hydroxyl groups in its structure, which contribute to its potent antioxidant capacities [14]. EGCG is a more effective radical scavenger than vitamins C and E [14,15]. Specifically, EGCG is known to have the ability to pass through the blood–brain barrier [16,17]. Thus, much attention has been paid to studying the role of EGCG in improving the cognitive function in various AD models [18,19,20].

However, few studies have assessed the effects of EGCG on *ex vivo* and *in vivo* models of scopolamine-induced amnesia, and the underlying mechanisms of EGCG to improve cognitive dysfunction need to be evaluated. The aim of this study was to investigate whether EGCG prevents scopolamine-induced memory impairment in an organotypic hippocampal tissue culture model and a male Sprague-Dawley (SD) rat model. The cognitive performance of rats was measured using three behavioral tests, namely the Y-maze, passive avoidance, and Morris water maze tests. AChE activity and the biomarkers of oxidative stress in hippocampi of the rats after the behavioral tests were also determined. Moreover, long-term potentiation (LTP) associated with learning and memory was analyzed using a multi-electrode array (MEA) in organotypic hippocampal slices of seven-day-old SD rats.

## 2. Materials and Methods

### 2.1. Reagents

Scopolamine hydrobromide, (−)-EGCG, Hank’s balanced salts (HBS), 4-(2-hydroxyethyl)-1-piperazineethanesulfonic acid (HEPES), polyethylenimine, 1,1,3,3-tetramethoxypropane, trichloroacetic acid (TCA), thiobarbituric acid (TBA), acetylthiocholine iodide (ATCI), and 5,5′-dithiobis(2-nitrobenzoic acid) (DTNB) were purchased from Sigma-Aldrich Co., LLC (St. Louis, MO, USA). Hank’s balanced salt solution (HBSS) and minimum essential medium (MEM) were purchased from Welgene, Inc. (Gyeongsan, Korea). Horse serum and penicillin–streptomycin were purchased from Biowest (Rue de la Caille, Nuaillé, France) and Gibco BRL (Grand Island, NY, USA), respectively. Lysis buffer was obtained from Noble Biosciences, Inc. (Hwaseong, Korea). Protease inhibitor was purchased from GenDEPOT (Barker, TX, USA). An EZ-SOD assay kit (DG-SOD400) was purchased from DoGenBio Co., Ltd. (Seoul, Korea). All reagents used in this experiment were of analytical grade.

### 2.2. Animals and Experimental Groups

**Figure 1:**
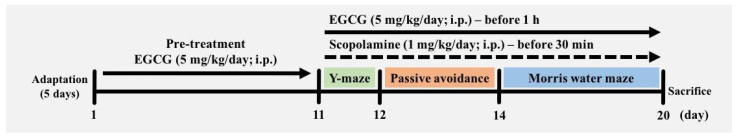
Diagram of the experimental schedule of behavioral tests in male Sprague-Dawley (SD) rats. EGCG: epigallocatechin gallate; i.p.: intraperitoneal injection.

Male SD rats (5 weeks old) were purchased from Saeron Bio Inc. (Uiwang, Korea). Rats were allowed to adapt to the environment for 5 days and were given free access to enough food and water *ad libitum*. The room condition was maintained with a 12/12 h light–dark cycle, 55% humidity, and a temperature of 22 ± 2 °C. All animal experiments were permitted by the Institutional Animal Care and Use Committee (KHUASP(GC)-18-040) of Kyung Hee University (Yongin, Korea), and were performed according to the guiding principles of the Council of the National Institutes of Health Guide for the Care and Use of Laboratory Animals. EGCG was administered by intraperitoneal injection (i.p.) at a dosage of 5 mg/kg body weight (BW)/day using the method of Gu et al. [21]. Scopolamine was administered by i.p. at a dosage of 1 mg/kg BW/day. Animals were randomly divided into four groups (six rats per group): control group (normal saline; i.p., as vehicle), SCOP group (scopolamine 1 mg/kg/day; i.p.), EGCG group (EGCG 5 mg/kg/day; i.p.), and EGCG + SCOP group (EGCG 5 mg/kg/day; i.p. + scopolamine 1 mg/kg/day; i.p.). As a pre-treatment, EGCG (5 mg/kg/day) was administered intraperitoneally to the EGCG and EGCG + SCOP groups for the first 10 days, whereas saline was administered intraperitoneally to the control and SCOP groups (Figure 1). In the behavioral tests conducted for another 9 days, EGCG and/or scopolamine at the same dosage were administered intraperitoneally daily to the rats of the SCOP, EGCG, and EGCG + SCOP groups 60 and 30 min before the beginning of the behavioral tests. For the control group, saline was administered intraperitoneally daily both 60 and 30 min before the behavioral tests, and for the EGCG group, saline was intraperitoneally administered daily 30 min before the behavioral tests. All experimental animals were sacrificed on the 20th day after three behavioral tests, namely the Y-maze, passive avoidance, and Morris water maze tests, were performed.

### 2.3. Behavioral Tests

#### 2.3.1. Y-Maze Test

A Y-type maze test was performed to examine short-term spatial working memory. The Y-type maze system was a structure in which three arms formed an alphabet Y-shape, and each arm was located 120° apart, with each arm having dimensions of length × height × width of 45 cm × 35 cm × 10 cm. Each arm of the Y-maze was randomly designated as region A, B, or C. For each animal, the order and the number of visits to the region of the arm were recorded for 10 min. When a rat entered each of three different consecutive arms, it was regarded as one alternation. The percentage of alternation was calculated as follows: (number of spontaneous alternations)/(total number of arm entries − 2) × 100. The entry and exit of each arm were recognized only when the rat’s hind legs passed more than half of the arm.

#### 2.3.2. Passive Avoidance Test

A passive avoidance test is a fear-aggravated test used to evaluate long-term avoidance memory. An apparatus was divided into light and dark compartments, with a gate between two compartments. An aluminum rod was placed on the bottom of a dark box, which caused an electric shock on the soles of the rats’ feet. Rats went through two separate trials, training for the acquisition of fear and a retention test to investigate whether the fear memory remained or not. In the training, rats were initially placed in the light chamber of the apparatus, then the middle partition was opened. When the rats moved to the dark room, the middle partition was closed, and an electric foot shock of 0.5 mA was applied once for 3 s. Then, the rats were placed back in their home cage. After 24 h, the retention test was performed to measure memory consolidation. Rats were placed in the light chamber, and the step-through latency time to enter the dark room was recorded for 5 min. If a rat remembered the previous exposure to the shock, it would avoid entering the dark chamber.

#### 2.3.3. Morris Water Maze Test

The Morris water maze test was designed to evaluate long-term spatial learning and memory by observing the escape latency, distance, speed, and time while rats are swimming in the water tank [22]. A circular water tank with a diameter of 180 cm and a height of 45 cm was filled with nontransparent water (23 ± 1 °C), and visual cues were placed on walls around the tank. A platform was submerged 1 cm below the surface of the water to prevent the platform from being seen. Training to find the submerged hidden platform continued for 5 days, with four daily sessions starting from different quadrants of the pool. Latency to reach the platform was measured, and after reaching the platform, rats were allowed to take a rest on the platform for 20 s. Rats that failed to find the platform within 60 s were guided to the platform and allowed to stay on the platform for 20 s. On the 6th day, in the probe test, rats were allowed to swim freely for 90 s in the platform-removed tank. All the movements of subjects were monitored by a video camera (SHC-650A; Samsung, Suwon, Korea) directly above the water tank. The swimming path and time were analyzed with the SMART video tracking system (SMART v3.0; Panlab, Barcelona, Spain).

### 2.4. Biochemical Assays

#### 2.4.1. Preparation of Hippocampus Homogenate

Under ether anesthesia, animals were decapitated after the behavioral tests, and the hippocampi of their brains were rapidly dissected and harvested. Hippocampal tissues were homogenized with lysis buffer containing phosphatase inhibitor. The homogenized tissues were centrifuged at 18,403× *g* for 20 min at 4 °C (PK121R; ALC International Srl, Cologno Monzese, Italy), and the supernatants were collected, immediately deep frozen, and kept at −70 °C prior to use.

#### 2.4.2. Measurement of Acetylcholinesterase (AChE) Activity

AChE activity was measured from the hippocampal homogenate harvested after the behavioral tests using the method of Ellman et al. [23]. In the method, AChE activity was measured using ATCI as a substrate. Specifically, 5 μL of sample and 65 μL of 50 mM sodium phosphate buffer (pH 7.4) were mixed and incubated for 15 min at 37 °C. Then, 70 μL of Ellman’s reaction mixture (0.5 mM of ATCI and 1 mM of DTNB in 50 mM sodium phosphate buffer (pH 7.4)) were added and incubated for 10 min at 37 °C. The mixtures were measured at 415 nm using a microplate reader (Infinite M200; Tecan Austria GmbH, Grödig, Austria). The results were obtained in the form of a percentage (%) relative to the activity of the control.

#### 2.4.3. Measurement of Superoxide Dismutase (SOD) Activity

SOD activity was measured from hippocampal homogenate harvested after the behavioral tests using a commercial EZ-SOD assay kit (DG-SOD400) according to the manufacturer’s instructions. Colorimetric reaction of the enzyme activity was measured at 450 nm using a microplate reader (Infinite M200). SOD activity of the hippocampus was expressed as a percentage (%) of inhibition using a blank with no sample input.

#### 2.4.4. Measurement of Malondialdehyde (MDA) Level

The level of lipid peroxidation was determined by reaction of MDA in hippocampal homogenates with TBA under acidic and thermal conditions. Hippocampus homogenate (100 µL) harvested after behavioral tests was mixed with 200 µL of a 10% TCA solution and then incubated for 15 min on ice. The reactants were centrifuged at 5000× *g* for 1 min to obtain the supernatants, followed by the addition of 200 µL of the supernatant or standard, and 200 µL of a 0.67% TBA solution. The mixtures were incubated at 100 °C for 10 min. After cooling, absorbance was measured at 531 nm using a microplate reader (Infinite M200). A standard curve was constructed by using 1,1,3,3-tetramethoxypropane.

### 2.5. Electrophysiological Experiments

#### 2.5.1. Organotypic Hippocampal Slice Culture

The organotypic hippocampal slice culture method was originally developed by Stoppini et al. [24]. Seven-day-old SD rats were decapitated, and the brains of their skulls were dissected quickly and carefully. The brain was immersed in ice-cold HBS-medium (HBS with 20 mM of HEPES). Then, the hippocampus of the brain was delicately dissected out and cut into 350 μm-thick slices using a McIlwain tissue chopper (Mickle Laboratory Engineering Co., Ltd., Gomshall, UK). Four or five slices were placed on a 0.4 μm membrane insert (Millicell-CM; Merck Millipore, Bedford, MA, USA), which was set into a 6-well plate filled with 1 mL of culture medium (50% of MEM, 25% of HBSS, 25% of horse serum, 5.25 g/L of *D*-glucose, 20 mM of HEPES, 1 mM of *L*-glutamine, and 1% of penicillin–streptomycin). The culture slices were incubated for 12–14 days before experimental treatments in an incubator (3111-TIF; Thermo Fisher Scientific, Waltham, MA, USA) controlled at 35 °C and 5% CO_2_.

#### 2.5.2. Preparation of Organotypic Hippocampal Slice Tissue on MEA

A hippocampal slice was carefully removed from a membrane insert and soaked completely in artificial cerebrospinal fluid (aCSF; 124 mM of NaCl, 26 mM of NaHCO_3_, 10 mM of *D*-glucose, 3 mM of KCl, 2 mM of CaCl_2_, 1 mM of MgCl_2_, and 20 mM of HEPES; pH 7.4). The slice was placed on an 8 × 8 MEA of 100 μm spacing between each 10 μm-diameter pre-coated with 0.01% polyethylenimine, followed by stabilization at 33 °C with 5% CO_2_ gas aeration at a flow rate of 3 mL/min of aCSF.

#### 2.5.3. Induction of LTP in Organotypic Hippocampal Slices

The MEA system was composed of an MEA, an amplifier (MEA1060), a stimulator (STG1004), and a temperature controller from Multi-Channel Systems (MCS GmbH, Reutlingen, Germany). The amplifier was placed in a grounded Faraday cage, and the MEA containing the hippocampal slice was transferred to the amplifier interface. A solution in the array was grounded using an Ag/AgCl pellet, and bipolar electrical stimulation was applied to the stratum radiatum CA2 region to stimulate the Schaffer collateral and commissural pathways. The intensity of bipolar test pulse stimulation was set at 100 mA, which was an optimized value to provide 40 to 65% of the maximum tissue response. High-frequency stimulation (HFS), which consisted of a total of 300 pulses with three trains of pulses delivered at 100 Hz and 5 min intervals, was applied to induce LTP. Each experiment was planned with a protocol totaling 95 min consisting of 30 min of test stimulation (one stimulation per min and including 10 min of baseline), immediately followed by 15 min of HFS and 50 min of test pulse stimulation. The aCSF was continuously circulated with a fresh one at a flow rate of 3 mL/min. Various concentrations (10, 50, and 100 μM) of EGCG were applied overnight prior to the beginning of the experiment by incubation. Scopolamine (300 μM) was applied along with aCSF for 85 min after 10 min of baseline recording during the experiments.

#### 2.5.4. Electrophysiology Data Processing

Unfiltered data were sampled from 60 recording channels at 25 kHz. Digitized analog MEA signals and isolated excitatory postsynaptic potentials were obtained from triggering amplitudes over 40 mV using the Recorder-Rack and MC Rack software (Multi-Channel Systems MCS). Peak-to-peak values between pre- and post-HFS conditions were compared to validate the stimulation protocol and provide a baseline over the same time frame.

### 2.6. Statistical Analysis

All data are expressed as the mean ± standard error of the mean (SEM). Statistical analysis was performed using SPSS software (Version 23.0; IBM SPSS Statistics Inc., Chicago, IL, USA). One-way analysis of variance, followed by a Tukey–Kramer honestly significant difference test, was performed to determine the significances of differences among means.

## 3. Results

### 3.1. Effects of EGCG on Short-Term Spatial Memory in the Y-Maze Test

**Figure 2:**
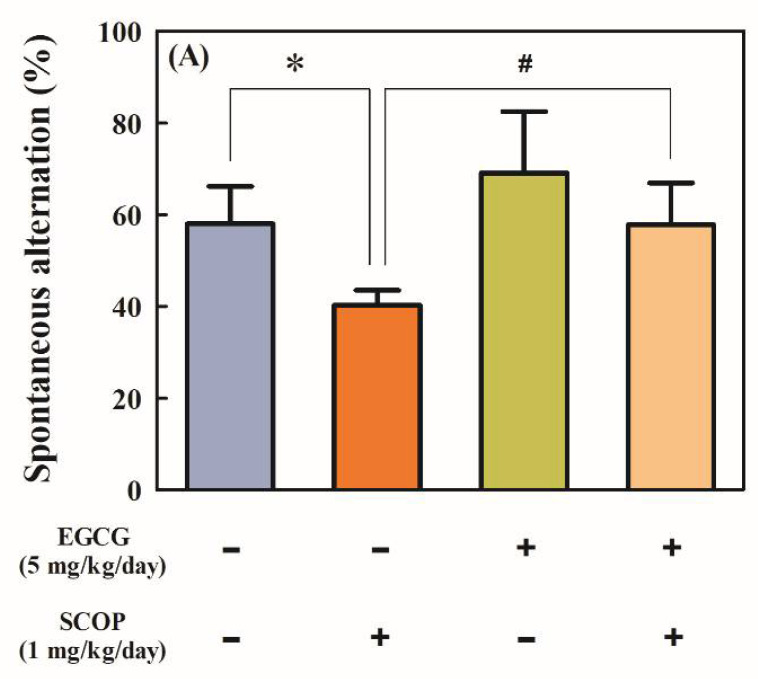

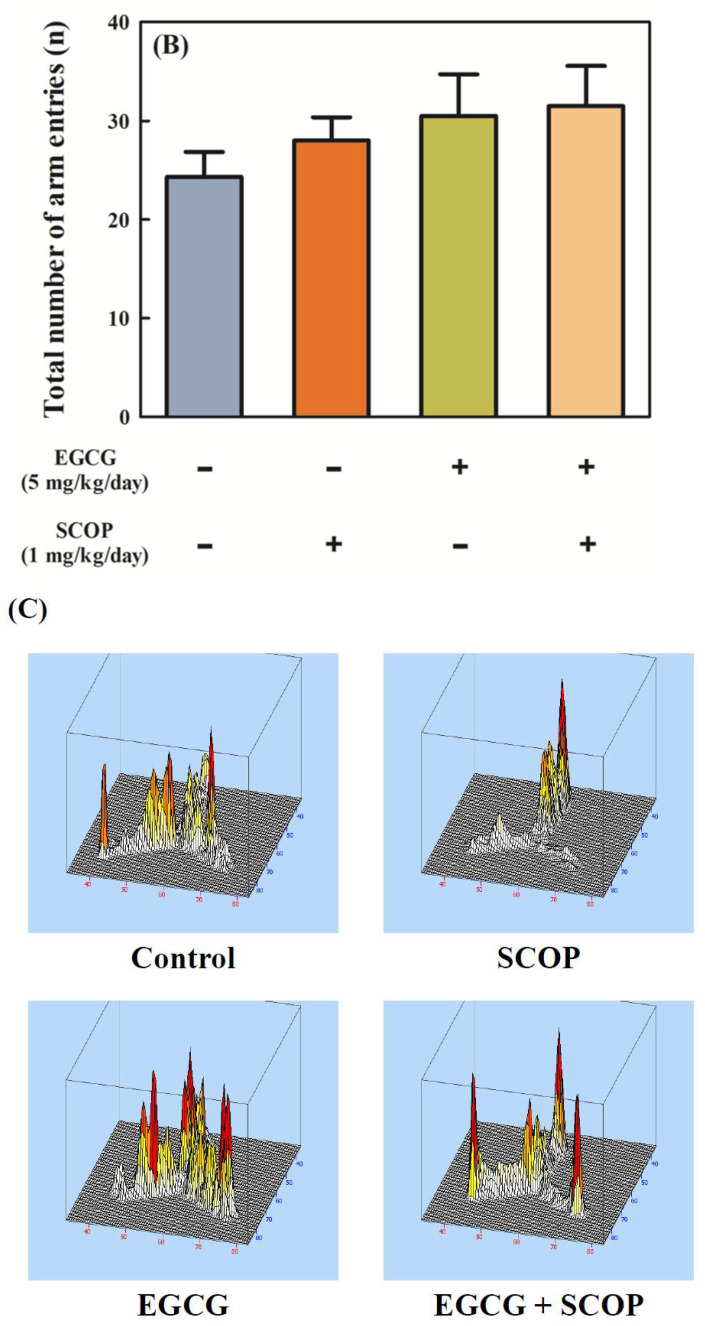
Effects of EGCG on (**A**) percentage of spontaneous alternation, (**B**) total number of arm entries, and (**C**) three-dimensional moving routes in the Y-maze test in SD rats against scopolamine (SCOP)-induced learning and memory dysfunction. Values represent the mean ± standard error of the mean (SEM; *n* = 6 in each group). * *p* < 0.05 (vs. control group), # *p* < 0.05 (vs. SCOP group) according to the Tukey–Kramer honestly significant difference test.

The SCOP group showed significantly (*p* < 0.05) impaired spontaneous alternation (40.3 ± 3.2%) compared with that of the control group (58.0 ± 3.3%) in the Y-maze test (Figure 2A). The EGCG group showed higher spontaneous alternation (69.1 ± 5.4%) compared with the control group, but there was no significant difference. The EGCG + SCOP group showed significant (*p* < 0.05) improvement of spontaneous alternation behavior (57.8 ± 3.7%) compared with the SCOP group. As shown in Figure 2B, there were no significant differences between all groups in the total number of arm entries. In the results of tracing the movement path of the rats in the Y-maze for 10 min (Figure 2C), the SCOP group showed irregular movements and uneven distribution compared with the control group. On the other hand, control and EGCG groups showed even distribution in each arm, and movements of the EGCG + SCOP group, which was pre-treated with EGCG in scopolamine-treated rats, were similar to those of the control and EGCG groups.

### 3.2. Effects of EGCG on Short-Term Learning and Memory in the Passive Avoidance Test

**Figure 3:**
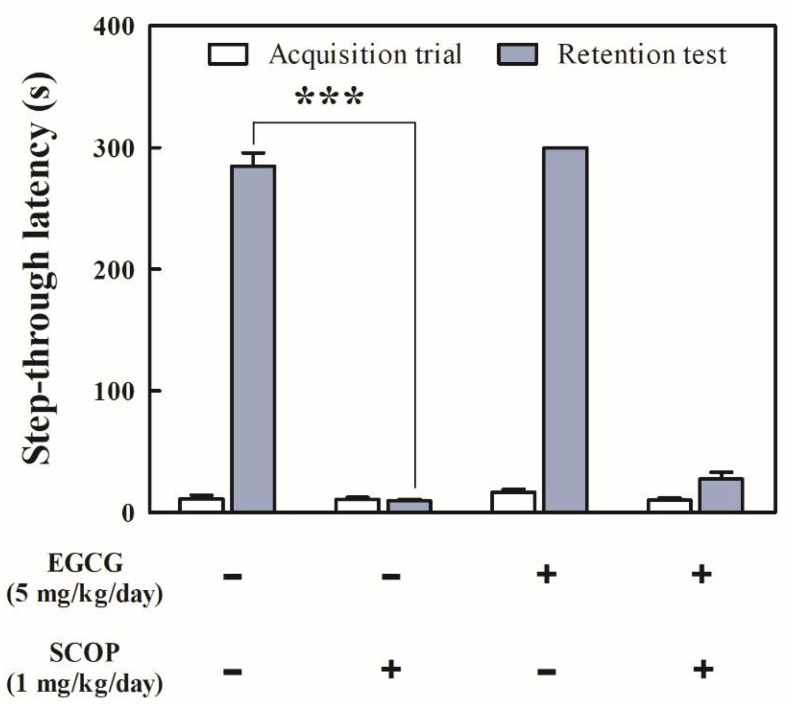
Effects of EGCG on step-through latency in the passive avoidance test in SD rats against SCOP-induced learning and memory dysfunction. Values represent the mean ± SEM (*n* = 6 in each group). *** *p* < 0.001 (vs. control group) according to the Tukey–Kramer honestly significant difference test.

There were no significant differences between the step-through latency of all experimental groups in the acquisition trial (Figure 3). In the retention test, the SCOP group (9.8 ± 1.1 s) had a short step-through latency time with a 96.6% decrease (*p* < 0.001) compared with that of the control group (284.6 ± 11.0 s). However, the EGCG + SCOP group had no significant increase in step-through latency time (27.8 ± 5.5 s) compared with the SCOP group.

### 3.3. Effects of EGCG on Long-Term Spatial Learning and Memory in the Morris Water Maze Test

**Figure 4:**
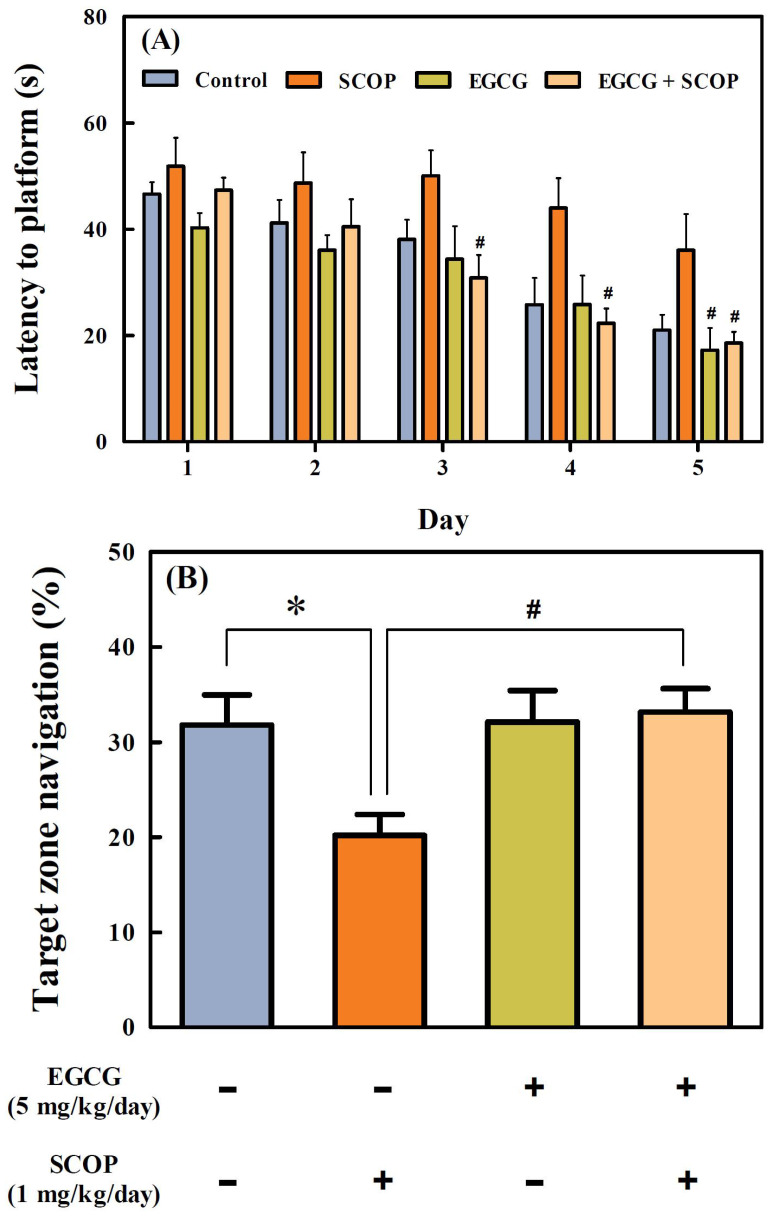

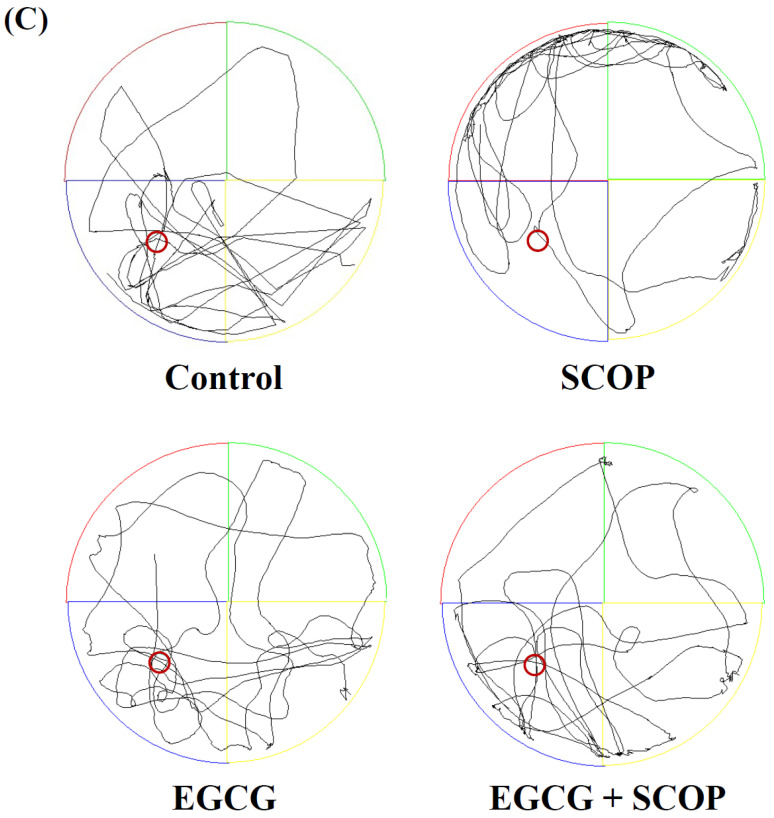
Effects of EGCG on (**A**) latency to platform during five consecutive training days, (**B**) percentage of swimming time in the target quadrant in probe test, and (**C**) swimming path tracing in probe test in the Morris water maze test in SD rats against SCOP-induced learning and memory dysfunction. Values represent the mean ± SEM (*n* = 6 in each group). * *p* < 0.05 (vs. control group), # *p* < 0.05 (vs. SCOP group) according to the Tukey–Kramer honestly significant difference test.

Escape latency gradually decreased in all groups for 5 days of training sessions (Figure 4A). The escape latency of the control and EGCG groups decreased from 46.6 ± 2.2 to 21.0 ± 2.9 s and from 40.3 ± 2.8 to 17.3 ± 4.2 s from day 1 to day 5, respectively. However, the escape latency of the SCOP group decreased from 51.9 ± 5.3 to 36.1 ± 6.9 s for 5 days, which was considerably higher than the other groups. On the other hand, the EGCG + SCOP group showed a decrease in escape latency from 47.3 ± 2.4 to 18.6 ± 2.1 s for 5 days and had a shorter escape latency than the SCOP group. Figure 4B shows the percentage of average swimming time in the target zone in which the platform was previously located in a probe test after 5 days of training. The percentage of swimming times in the target zone of the SCOP group (20.2 ± 2.2%) was significantly lower (*p* < 0.05) than the control group (31.8 ± 3.2%). The EGCG group (32.1 ± 3.3%) showed swimming time in the target zone similar to the control group. The EGCG + SCOP group (33.2 ± 2.5%) significantly (*p* < 0.05) increased the swimming time in the target zone compared with the SCOP group as negative control group treated with scopolamine only. In addition, Figure 4C shows swimming patterns of rats in the probe test for 90 s. The SCOP group stayed longer in the other quadrants than the target zone, whereas the control and EGCG groups spent relatively more time in the target zone. The EGCG + SCOP group showed similar swimming patterns to the control and EGCG groups.

### 3.4. Effects of EGCG on Cholinergic Function in the Hippocampus

**Figure 5:**
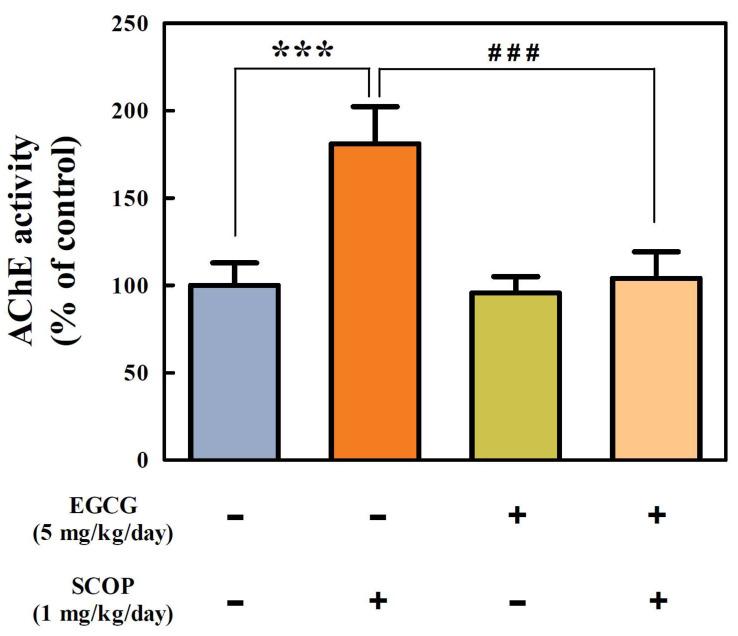
Effects of EGCG on acetylcholinesterase (AChE) activity in the hippocampus in SD rats against SCOP-induced learning and memory dysfunction. Values represent the mean ± SEM (*n* = 6 in each group). *** *p* < 0.001 (vs. control group), ### *p* < 0.001 (vs. SCOP group) according to the Tukey–Kramer honestly significant difference test.

There was a significant (*p* < 0.001) increase in the AChE activity (181.0 ± 8.7%) of hippocampal homogenate in the SCOP group compared with the control group (100%) (Figure 5). However, the EGCG + SCOP group (103.9 ± 6.3%) significantly (*p* < 0.001) inhibited the increase in AChE activity compared with the SCOP group induced by scopolamine. The EGCG group showed 95.7 ± 9.3% of AChE activity.

### 3.5. Effects of EGCG on SOD Activity and MDA Level in the Hippocampus

**Figure 6:**
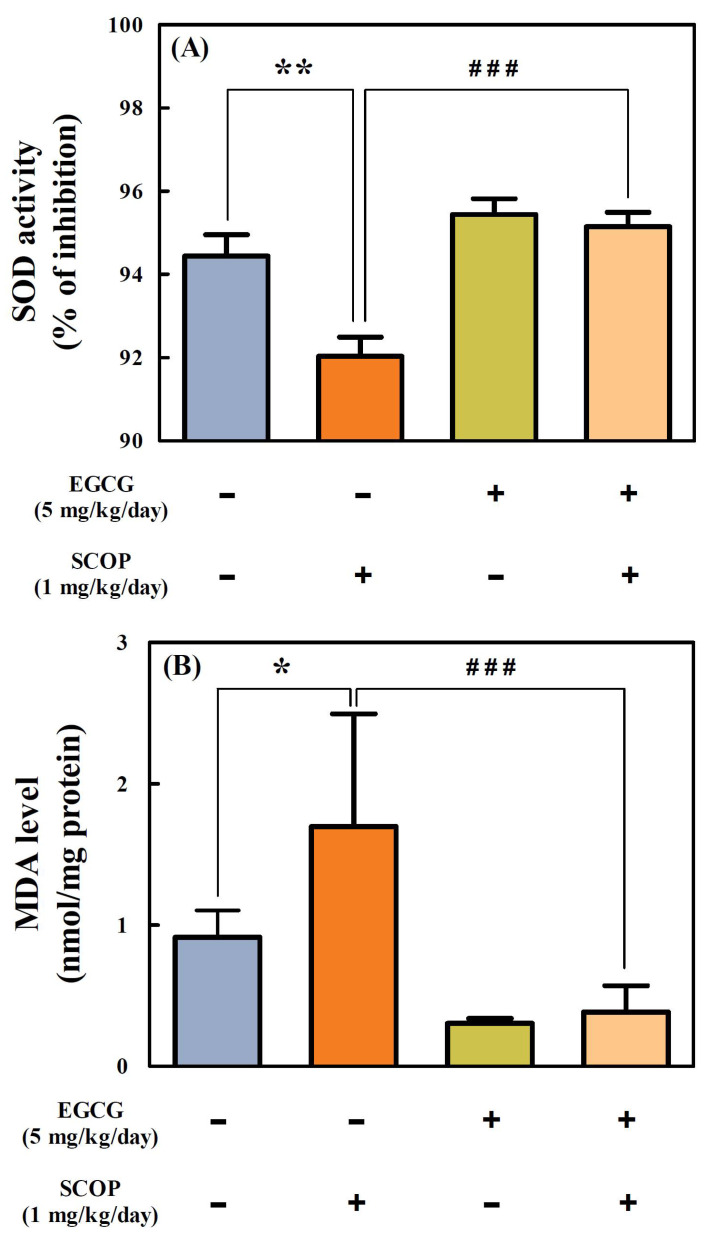
Effects of EGCG on (**A**) superoxide dismutase (SOD) activity and (**B**) malondialdehyde (MDA) level in the hippocampus in SD rats against SCOP-induced learning and memory dysfunction. Values represent the mean ± SEM (*n* = 6 in each group). * *p* < 0.05, ** *p* < 0.01 (vs. control group), ### *p* < 0.001 (vs. SCOP group) according to the Tukey–Kramer honestly significant difference test.

SOD activity (92.0 ± 0.5%) of the hippocampal homogenate in the SCOP group was significantly (*p* < 0.01) suppressed compared with the control group (94.4 ± 0.5%) (Figure 6A). However, the EGCG + SCOP group showed a significant (*p* < 0.001) increase in the SOD activity (95.1 ± 0.3%) of hippocampal homogenate as compared with the SCOP group. The SOD activity in the EGCG group was 95.4 ± 0.9%.

The MDA level in the hippocampus of the SCOP group (1.70 ± 0.8 nmol/mg protein) showed a significant (*p* < 0.05) increase compared with the control group (0.91 ± 0.19 nmol/mg protein) (Figure 6B). However, the MDA level (0.38 ± 0.19 nmol/mg protein) in the EGCG + SCOP group showed a significant (*p* < 0.001) decrease compared with the SCOP group. The EGCG group had an MDA level of 0.30 ± 0.03 nmol/mg protein.

### 3.6. Effects of EGCG on LTP in Organotypic Hippocampal Slices

**Figure 7:**
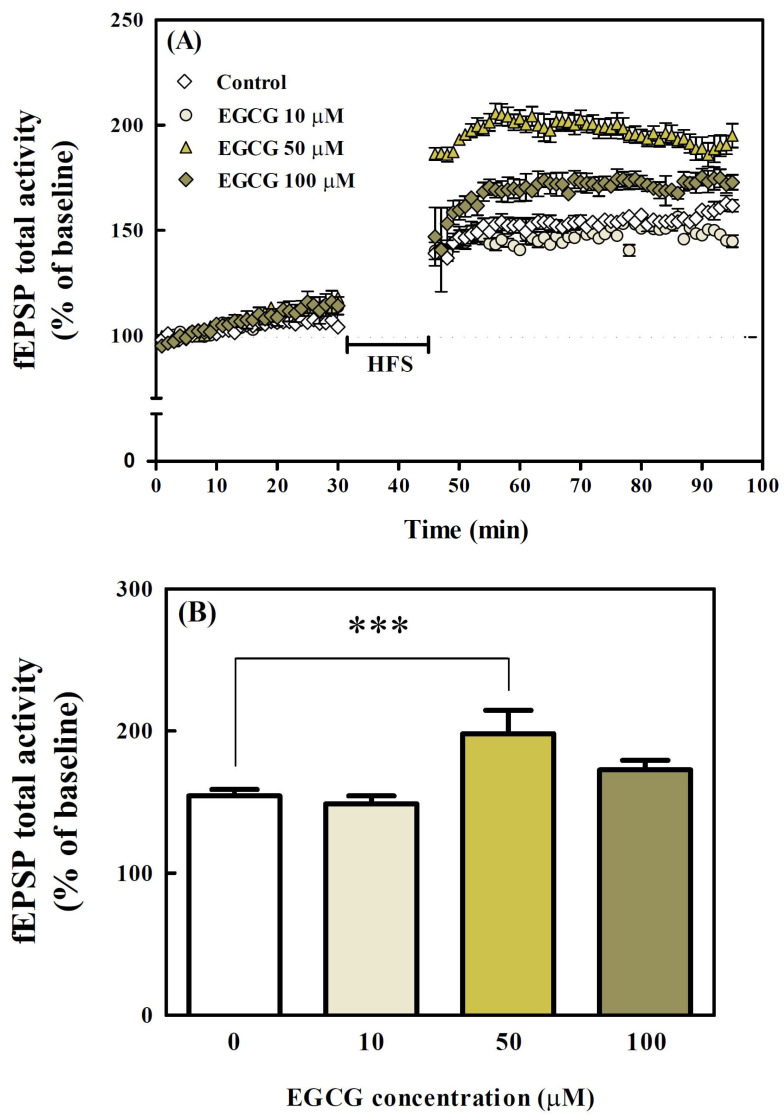

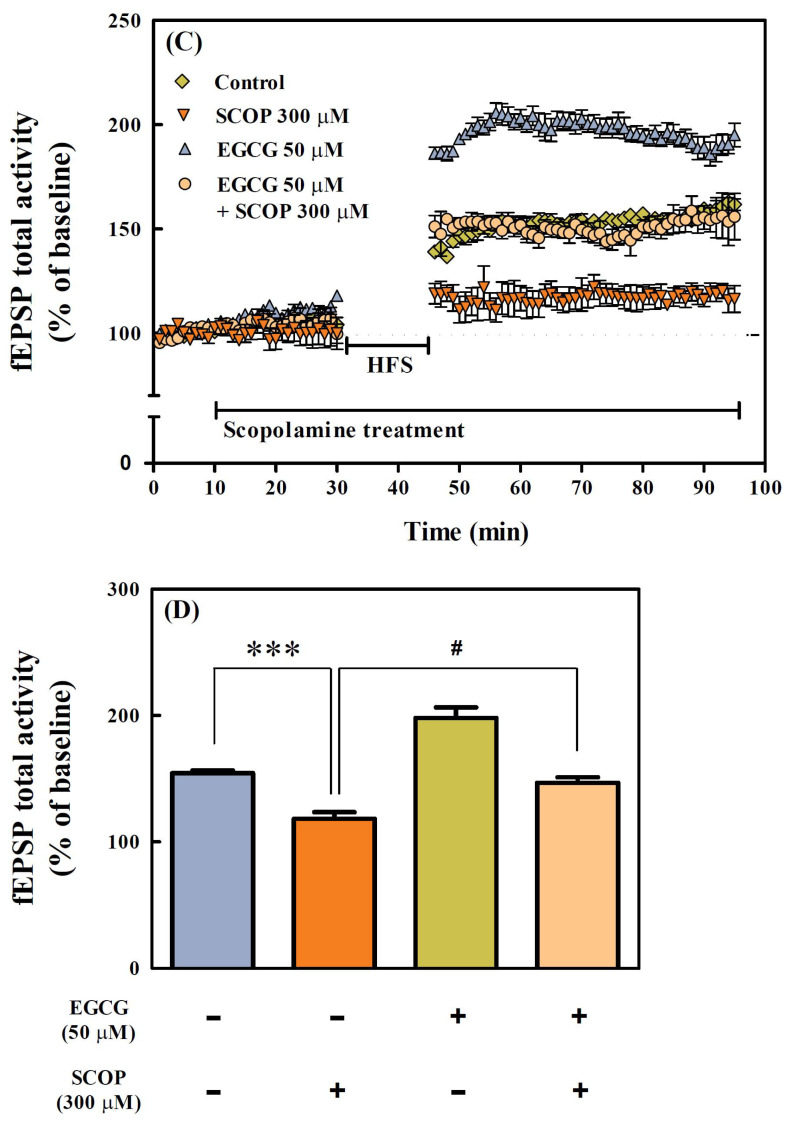
Effects of EGCG on long-term potentiation (LTP) in organotypic hippocampal slices from the brains of seven-day-old SD rats. (**A**) Field excitatory postsynaptic potential (fEPSP) total activity slope (% of baseline) before and after high-frequency stimulation (HFS) in organotypic hippocampal slices treated with various concentrations (10, 50, and 100 µM) of EGCG. (**B**) Average LTP amplitude measured at 30–40 min after HFS. (**C**) The fEPSP total activity slope (% of baseline) before and after HFS in organotypic hippocampal slices treated with EGCG (50 µM) and SCOP (300 µM). (**D**) Average LTP amplitude measured at 30–40 min after HFS. Values represent the mean ± SEM (*n* = 4 in each group). *** *p* < 0.001 (vs. control group), # *p* < 0.05 (vs. SCOP group) according to the Tukey–Kramer honestly significant difference test.

Effects of EGCG on LTP in the hippocampal CA1 region were measured by recording field excitatory postsynaptic potential (fEPSP) using MEA. In the control group, the fEPSP reached 154.3 ± 2.3% (Figure 7A,B). The group treated with 10 μM of EGCG had a similar level (148.7 ± 3.2%) to the fEPSP of the control group. There was no significant difference between the control group and the 10 μM and 100 μM of EGCG-treated groups. However, the 50 μM of EGCG-treated group had an fEPSP value of 199.0 ± 8.4%, which was significantly (*p* < 0.001) different from the control group.

Furthermore, the effects of EGCG on scopolamine-induced LTP impairment were investigated (Figure 7C,D). In the SCOP group, the fEPSP was significantly (*p* < 0.001) decreased to 118.3 ± 5.2% by 300 μM scopolamine-treatment compared with the control group. However, when scopolamine was co-treated to the hippocampal slices pre-treated with 50 μM of EGCG (EGCG + SCOP group), fEPSP significantly (*p* < 0.05) increased (146.7 ± 4.3%) to a level similar to the control group.

## 4. Discussion

Neurodegenerative diseases such as AD cause behavioral and cognitive dysfunction and are major health problems that increase financial burdens in aging and aged societies [1]. Natural therapeutic agents, including polyphenols, have recently received attention as an alternative medicine for neurodegenerative diseases [25]. In the present study, EGCG, a natural green tea bioactive polyphenol, alleviated the cognitive deficits in scopolamine-induced amnestic SD rats through behavioral tests. EGCG attenuated the blockade of LTP in organotypic hippocampal slices from seven-day-old SD rats. EGCG increased SOD activity, reduced AChE activity, and decreased MDA level in the hippocampus. These results suggested that EGCG may be useful as a potential material for improving the cognitive impairments.

Scopolamine, a well-known anticholinergic drug, is commonly used as an experimental standard drug to induce cognitive impairment in *in vivo* models [7,26]. It was previously reported that administration of scopolamine resulted in a lack of cognitive performance in the radial maze and interfered with acquisition in the Morris water maze test [5]. To confirm the attenuating effects of EGCG against scopolamine-induced learning and memory impairment in male amnestic SD rats, the Y-maze, passive avoidance, and Morris water maze tests were conducted in this study.

Performance in the Y-maze test is related to short-term spatial memory. Rats prefer exploring a new arm rather than returning to the previously visited arm in the Y-maze, which is due to the instinct of rodents to explore new environments [27]. In the present study, EGCG administration (5 mg/kg/day) with and without scopolamine significantly (*p* < 0.01) increased spontaneous alternation behavior in SD rats compared with those treated with scopolamine only. The animals showed a similar total number of arm entries, indicating that administration of scopolamine and EGCG did not alter spontaneous locomotor activity, but affected memory only. This was in accordance with previous studies that excluded the possibility of scopolamine interfering with locomotor activity in the memory test [28,29]. These results from Y-maze tests in the male SD rats indicate that EGCG inhibits short-term spatial memory deterioration.

Passive avoidance tests are based on the conflict between fear and preference for darkness and are performed to evaluate short-term learning and memory abilities in rats that are exposed to an unavoidable electric shock when entering a dark compartment [30]. In this study, the SCOP group did not significantly remember receiving the electrical shock from the day before, and entered the dark compartment almost immediately. However, EGCG treatment considerably retarded the step-through latency. Administration of EGCG effectively alleviated the reduction of step-through latency induced using scopolamine. The passive avoidance test results above indicate that EGCG inhibits long-term avoidance memory impairments in the SD rats.

The Morris water maze test is a widely accepted method to evaluate the long-term spatial learning and memory of animals in the laboratory [31]. In the training trials, the latency to find the platform indicates how quickly they can learn and memorize the location of the hidden platform, evaluating long-term spatial learning capabilities. In this study, the escape latency to the platform of all groups gradually decreased over five days, but the SCOP group had a slower learning rate than the other groups. After the training, the swimming time in the target zone where the platform was located was measured to evaluate the spatial memory capability of rats. The SCOP group was swimming randomly and irregularly in the maze, and the swimming time of the rats crossing the target zone was significantly (*p* < 0.05) decreased compared with that of the control group. The deterioration performance of the SCOP group implied marked long-term learning and memory impairment in SD rats induced by scopolamine. The EGCG + SCOP group performed accurate navigation to the target quadrant and showed similar swimming time in the target zone compared with the control group. These results of the Morris water maze test suggest that EGCG effectively attenuates long-term spatial learning and memory deficits.

Overall, administration of EGCG effectively improved learning and memory dysfunction in SD rats induced by scopolamine. The results from three different behavioral tests (Y-maze, passive avoidance, and Morris water maze tests) were similar to those of previous studies that have reported improvement of learning and memory impaired by scopolamine [9,28,29]. The formation of learning and memory in these behavioral experiments is highly related to the hippocampus. Hippocampal lesions cause performance impairment in Y-maze alternation, suggesting that the spatial (i.e., current location) and non-spatial (i.e., intended destination) aspects of the behavior of rats are encoded by the hippocampal neurons [32]. A correlation between the failure of spatial navigation in neurodegeneration and specific patterns of neural damage involving the hippocampus indicates that the hippocampus is also very important for spatial memory and navigation [33].

Cholinergic deficit is one of the most prominent mechanisms of AD that is associated with deterioration of cognitive functions, including memory loss [34]. Cholinergic transmission is terminated mainly by AChE, an enzyme responsible for the degradation of ACh, an important neurotransmitter, in the cholinergic neurons [35]. Scopolamine is known to induce an increase in AChE activity [7]. AChE inhibitors have been reported to improve cognitive function in scopolamine-treated rats [7,34]. In this study, the administration of EGCG inhibited the increase of AChE activity by scopolamine in the hippocampus harvested after the behavioral tests. It has been reported that decreased AChE activity in the brain is associated with improved cognitive function [29,36]. Thus, these results in this study suggest that EGCG effectively improves learning and memory function partly due to inhibition of AChE activity in the hippocampus.

Oxidative stress causes brain tissue damage, contributing to learning and memory deficits [37]. Aerobic organisms have a wide range of endogenous antioxidant defense systems, embracing enzymatic and non-enzymatic antioxidants to protect tissue from oxidative damage [38]. SOD is an antioxidant enzyme that regulates oxidative stress by removing superoxide radical anions. MDA, a byproduct of lipid peroxidation, is an indicator of oxidative stress.

In this study, the activity of antioxidant enzyme SOD was significantly (*p* < 0.01) suppressed in the SCOP group compared with the control group treated without EGCG or scopolamine. In addition, the administration of scopolamine significantly (*p* < 0.05) increased the level of lipid peroxidation in the hippocampus of the SD rat brain compared with the control. However, pre-administration of EGCG in rats inhibited a scopolamine-induced decrease in SOD activity and an increase in MDA in their hippocampi. Similar to the above results of this study, it was previously reported that cognitive function was improved by the neuroprotective effects due to the reduction of oxidative stress by increasing SOD activity and decreasing MDA levels [39,40]. Thus, the anti-amnesic effects of EGCG in *in vivo* behavioral tests can be demonstrated by the neuroprotective effects of EGCG, which was used in this study as an exogenous antioxidant to support the antioxidant defense system and prevent oxidative damage in the neuron.

LTP provides a persistent increase in the efficiency of synaptic transmission of neurons in the hippocampus [41]. LTP is one of the major cellular mechanisms of learning and memory formation in the hippocampus, and is widely accepted as a major experimental model of AD [42]. An irreversible AChE inhibitor (diisopropyl fluorophosphate) has been reported to produce a hippocampal LTP facilitation that is mediated by direct activation of the cell-surface and intracellular M1-muscarinic ACh receptors via endogenous ACh, showing the association of AChE inhibition with LTP enhancement [43].

The administration of scopolamine in organotypic hippocampal slices from seven-day-old SD rats impaired the LTP in CA1 region neurons, suggesting that the impairment of LTP in the SCOP group led to cognitive decline in rats, which was similar to the results reported in previous studies [44,45]. However, long-term treatment with EGCG significantly (*p* < 0.05) attenuated scopolamine-induced blockade of LTP in the organotypic hippocampal slices from seven-day-old SD rats. Similar to these results, the administration of *p*-coumaric acid and diosmin has been reported to reverse LTP impairment induced by scopolamine, and showed a cognitive recovery effect in behavioral tests [46,47]. These findings in our study suggest that improvement of cognitive performance during the behavioral tests in the EGCG-pretreated group before scopolamine injection was associated with LTP enhancement.

## 5. Conclusions

In the present study, the anti-amnesic effects of a major green tea flavan-3-ol, EGCG, on cognitive deficits were evaluated with *in vivo* and *ex vivo* models. In behavioral studies, EGCG inhibited impairment of short-term working memory, long-term avoidance memory, and long-term spatial memory in scopolamine-induced learning- and memory-deficit rats. EGCG decreased AChE activity and MDA level, but increased SOD activity in hippocampi of the SD rats after the behavioral tests. EGCG enhanced synaptic plasticity in the hippocampus dissected out of the brain in the seven-day-old SD rats. These findings demonstrated the potential of EGCG as a therapeutic agent for amnesia. Furthermore, daily consumption of food products such as green tea rich in EGCG may be beneficial to inhibit cognitive dysfunction in neurodegenerative diseases such as AD.

## Data Availability

The data are contained within the article.
